# URGCP/URG4 promotes apoptotic resistance in bladder cancer cells by activating NF-κB signaling

**DOI:** 10.18632/oncotarget.5134

**Published:** 2015-09-02

**Authors:** Minglong Wu, Junxing Chen, Yuxi Wang, Jinqian Hu, Chang Liu, Chunxiang Feng, Xiaoyong Zeng

**Affiliations:** ^1^ Department of Surgery, Tongji Hospital, Tongji Medical College, Huazhong University of Science and Technology, Wuhan, Hubei, China; ^2^ Department of Urology, Tongji Hospital, Tongji Medical College, Huazhong University of Science and Technology, Wuhan, Hubei, China; ^3^ Department of Urology, the First Affiliated Hospital, Sun Yat-Sen University, Guangzhou, Guangdong, China

**Keywords:** bladder cancer, URGCP/URG4, apoptosis, NF-κB

## Abstract

Cisplatin is a well-known chemotherapeutic agent, it could cause DNA damage and induce apoptotic cell death, but the cisplatin resistance also appears, it's important to reveal the mechanisms of cisplatin resistance [1]. URGCP/URG4 is overexpressed in various tumors and plays critical role during tumor development. We found URGCP/URG4 was upregulated in bladder cancer cells and tissues, URGCP/URG4 overexpression increased the resistance to cisplatin-induced apoptosis in bladder cancer, and promoted anti-apoptotic genes expression, such as Bcl-2, Survivin, MCL-1, FLIP, and downregulated Caspase-3 expression, Knockdown of URGCP/URG4 decreased the resistance to cisplatin-induced apoptosis, and inhibited anti-apoptotic genes expression, such as Bcl-2, Survivin, MCL-1, FLIP, and upregulated Caspase-3 expression. Mechanism analysis found URGCP/URG4 activated NF-κB pathway which is a well-known anti-apoptotic pathway and promoted the expression of NF-κB targeted genes. So we speculated URGCP/URG4 regulates cisplatin-induced apoptosis by activating NF-κB pathway. We also analyzed the correlation between URGCP/URG4 expression and clinical clinicopathologic, and found its expression was positively correlated with bladder cancer progression, it can serve as a valuable prognostic factor. In summary, URGCP/URG4 promotes the resistance to cisplatin-induced apoptosis by activating NF-κB pathway, and is an unfavorable prognostic factor for bladder cancer.

## INTRODUCTION

Bladder cancer, one of the most common cancer types in the urinary system arising from mucous membrane of urinary bladder, accounts for 3% of all malignant tumors all around the world and over 350,000 people are diagnosed each year [2]. It is the 7th most common cancer in men and the 17th most common cancer in women in the world [3]. Like other tumors, bladder cancer cells often have faulty apoptotic pathways, performing an anti-apoptotic effect which leads to the resistance to the apoptosis-inducing cancer treatment, such as radiation or chemotherapy [4]. Consistent with accumulating studies show that apoptosis plays an important role in both carcinogenesis and cancer treatment, the mechanism of anti-apoptotic effect in tumors is being paid more attentions recently.

Upregulator of cell proliferation (URGCP), also known as upregulated gene-4 (URGCP/URG4), is a novel gene which is located on 7 chromosome (7p13). In hepatocellular carcinoma (HCC), URGCP/URG4 may be considered as a natural effector of hepatitis Bx antigen (HBxAg) which contributes to hepatocarcinogenesis, URGCP/URG4 is up-regulated in the presence of HBxAg. Overexpression of URGCP/URG4 not only promotes the growth and survival of HCC *in vitro*, but also accelerates the development of HCC *in vivo* [5]. URGCP/URG4 is upregulated in human gastric cancer tissues and cell lines, and overexpression of URGCP/URG4 promotes gastric cancer cells proliferation and tumorigenicity [6]. Moreover, URGCP/URG4 can function as a proto-oncogene and is associated with tumor metastasis and recurrence in osteosarcoma [7]. These studies suggest that URGCP/URG4 promotes the progression and development of various tumors. However, the role of URGCP/URG4 in bladder cancer has not been elucidated.

Here, we first determined URGCP/URG4 expression in bladder cancer cells and tissues, then we studied the effect of URGCP/URG4 on anti-apoptotic effect by modulating its expression both *in vitro* and *in vivo*. We also studied the mechanism of its anti-apoptotic effect in bladder cancer. Finally, we analyzed the relationship between URGCP/URG4 expression and clinicopathplogic characteristics to examine whether URGCP/URG4 could serve as an unfavorable prognostic factors.

## MATERIALS AND METHODS

### Patients and tissue specimens

Eight fresh bladder tumor tissues and one normal bladder tissue were collected from the Department of Urology, Tongji Medical College, Huazhong University of Science and Technology. The tissues were snap frozen immediately and stored in liquid nitrogen until further use. A cohort of 172 paraffin-embedded bladder cancer samples, which also were hisotologically and clinically diagnosed from the Department of Urology, Tongji Medical College, Huazhong University of Science and Technology, between 1996 and 2010. For the research proposes using these samples. Prior patient's consent and approval from the ethics committee of Huazhong University of Science and Technology were obtained. The detailed clinicopathological information is shown in Table 1.

**Table 1:** Clinicopathological characteristics of patient samples and expression of URG4 in bladder cancer

Characteristics	Number of cases (%)
**Age (years)**	
<64	81(47.1)
≥64	91(52.9)
**Gender**	
Male	157(91.2)
Female	15(8.8)
**Degree of differentiation**	
I	32(18.6)
II	53(30.8)
III	87(50.6)
**T classification**	
T_1_	90(52.3)
T_2_	82(47.7)
**N classification**	
N_0_	150(87.2)
N_1_	22(12.8)
**Number**	
<3	108(62.8)
≥3	64(37.2)
**Vital status (at follow-up)**	
alive	67(39.0)
Dead	105(61.0)
**Expression of URG4**	
Low expression	66(38.4)
High expression	106(61.6)
**Size**	
<3	72(41.9)
≥3	100(58.4)
**Recurrence**	
Yes	82(47.7)
No	90(52.3)

### Cell culture

The human bladder tumor cell line 5637 (from an epithelial grade II carcinoma) and the human bladder tumor cell line RT4 (from a transitional cell papilloma) were purchased from the American Type Culture Collection (Rockville, MD). Cells were cultured in RPMI 1640 medium (Gibco, Grand Island, NY, USA), supplemented with 10% fetal bovine serum (Gibco), 2 mM L-glutamine (Gibco), 100 μM nonessential amino acids (NEAA), 50U/mol penicillin, 50 mg/ml streptomycin. The primary-cultured normal bladder epithelial cells were used as the normal control (represented as NC), which were cultured in RPMI 1640 medium, supplemented with 5 ng/mL epithelial growth factor, 70 ng/mL phosphoethanolamine, and 10% fetal bovine serum. Cisplatin (Sigma, Saint Louis, MO, USA) was used to treat bladder cancer cells at concentration of 40 μg/ml, for 24 h.

### Plasmid construction and retroviral infection

To construct the overexpression plasmid, The CDS sequence of URGCP/URG4 was PCR amplified from cDNA of primary-cultured normal bladder epithelial cells, and cloned into the pMSCV-retro-puro vector (Clontech). The following primers were used: forward primer: 5′-CCAGATCTACCATGG CGTCGCCCGGGCATTC-3′ and reverse primer: 5′-GCCGAATTCTCACAGCCGTCTCACCAGCT-3′. To knock-down URGCP/URG4, a human siRNA sequence was cloned into the pSUPER. retro. puro plasmid (OligoEngine) to generate pSUPER. retro. URGCP/URG4-RNAi, the siRNA sequence was: ACCAAAGACTTGCCCTGGAATT, which was synthesized by Invitrogen. Retroviral production and infection were performed as described previously [8]. Stable cell lines expressing URGCP/URG4 or silencing URGCP/URG4 were selected using puromycin. The URGCP/URG4 expression was confirmed by immunoblotting.

### Apoptosis analysis by terminal deoxynucleotidyl transferase-mediated dUTP nick end labeling (TUNEL)

Apoptosis assay of Cells and tissues samples was determined by TUNEL used an *In Situ* Cell Death Detection Kit, Fluorescein (Roche Applied Science, South San Francisco, CA, USA) according to the manufacturer's protocol. The number of TUNEL-positive cells was counted under a fluorescence microscope. The percentages of apoptotic cells were calculated from the ratio of apoptotic cells to total cells counted. Tissue sections were counter-stained with hematoxylin. Mount and observe sections under light microscopy. The experiment was performed for independently three times for each cell line.

### Apoptosis analysis by flow cytometry

Cells were harvested by trypsinization, washed three times in phosphate buffered saline (PBS), and resuspended in 0.5 ml PBS. Propidium iodide (PI) and a fluorescein isothiocyanate (FITC)-conjugated monoclonal antibody specific for Annexin V (Sigma) were incubated with the cells at 4°C for 30 min. Cell apoptosis was measured using flow cytometry (Becton Dickinson Biosciences, San Jose, CA) and the data were analyzed by ModFit LT software package. The experiment was performed for independently three times for each cell line.

### Western blotting, immunohistochemistry (IHC) and immunofluorescence

Western blotting assay was performed according the method reported previously [9], using anti-URGCP/URG4 (Sigma, HPA02134), anti-p65 (Abcam, ab7970), anti-p84 (Epitomics, EPR5662 (2)), anti-eEF1A1+eEF1A2+eEF1AL3 (Abcam, ab37969, we indicated this antibody as EF-1α), anti-Bcl-2 (Abcam, ab77567) and anti-Caspase-3 antibodies (Abcam, ab17868). The membranes were stripped and re-probed with anti-alpha-Tubulin antibody (Abcam) for a loading control. The protocol of Immunohistochemistry in clinical samples was performed according to previous report [10]. Anti-URGCP/URG4 antibody (Sigma, HPA02134) was used. The tissue sections were reviewed and scored by two pathologists independently. Immunofluorescence also was performed according to previous report [11], anti-p65 antibody (Abcam, ab7970) was used.

### Luciferase reporter assay for NF-κB transcriptional activity

3 × 10^4^ cells per well were seeded in triplicates in 24-well plates and were allowed to settle. One hundred nanograms of pNF-κB-luciferase plasmid or control-luciferase plasmid plus 10 ng pRL-TK renilla plasmid (Promega) were transfected into bladder cancer cells using the Lipofectamine 2000 reagent (Invitrogen, Carlsbad, CA, USA), according to the manufacturer's instruction. Luciferase and renilla signals were measured 48 hours after transfection using the Dual Luciferase Reporter Assay Kit (Promega) according to a protocol provided by the manufacturer.

### RNA extraction and quantitative real-time PCR (qRT-PCR)

Total cellular RNA was extracted using the Trizol reagent (Invitrogen) according to a protocol provided by the manufacturer. The qRT-PCR reactions were performed using dye SYBR Green I (Molecular Probes, Invitrogen) with initial denaturation at 95°C for 10 min followed by 40 cycles at 95°C for 20 s, 60°C for 30 s and 72°C for 1 min. Gene expression levels were quantified using the 7500 Fast Real Time Sequence detection system Software (Applied Biosystems, Foster City, CA). The expression of the genes were defined based on the threshold cycle (Ct), and Glyceraldehyde 3-phosphate dehydrogenase (*GAPDH*) was used as reference gene that acts as an internal reference to normalize the mRNA expression, and calculated as 2^−[(Ct of *CCND1, BcL-2, MMP9, VEGF*) − (Ct of *GAPDH*)]^.

The primers used were:

*URGCP/URG4* forward: 5′-CTTCATCCTGAGTC CCTACCG-3′,

*URGCP/URG4* reverse: 5′-GCCGT TCTGCTGC ATTCG-3′;

*CCND1* forward: 5′-AACTACCTGGACCGCTTC CT-3′,

*CCND1* reverse: 5′-CCACTT GAGCTTGTTCA CCA-3′;

*BcL-2* forward: 5′-CATGCTGGGGCCGTACAG-3′,

*BcL-2* reverse: 5′-GAACCGGCACCTGCACAC-3′;

*MMP9* forward: 5′-ACGACGTCTTCCAGTAC CGA-3′,

*MMP9* reverse: 5′-TTGGTCCACCTGGTTCAA CT-3′;

*VEGF* forward: 5′-GTGTCCAGTGTAGATGAA CTC-3′,

*VEGF* reverse: 5′-ATCTGTAGACGGACACACA TG-3′;

*GAPDH* forward: 5′-GACTCATGACC ACAGTCC ATGC-3′,

*GAPDH* reverse: 3′-AGAGGCAGGGATGATGT TCTG-5′.

### Mouse model

5-week-old BALB/c nude mice were used for the bladder cancer cell lines RT4 xenograft model. Medium (0.2 ml) containing 5 × 10^6^ RT4 was injected subcutaneously into the left and right posterior flank regions of each mouse. Mice were housed in a pathogen-free environment and tumor growth was examined every three days. After the tumor growing for 20 days, animals were assigned to *i.p*. injection of cisplatin (20mg/kg), on following days 24, 28, 32, 36, and 40. Animals were euthanized and tumors were excised, weighed, and subjected to pathologic examination. All animal experiments were performed in accordance with the Guide for the Care and Use of Laboratory Animals and conformed by Tongji Medical College, Huazhong University of Science and Technology ethical guidelines for animal experiments.

### Statistical analyses

All statistical analyses were carried out using the SPSS version 13.0 software (SPSS, Chicago, IL, USA). The Chi-Square test was used to assess the correlation between URGCP/URG4 expression and clinicopathological characteristics. Bivariate correlations between the study variables were examined using Spearman's rank correlation coefficients. Relative risks of death associated with URGCP/URG4 expression and other predictive variables were estimated through univariate and multivariate Cox-regression analysis. Kaplan-Meier method was used to plot overall survival curves, the log-rank test was used to compare the survival curves. A two-tailed paired student's *t* test was used to evaluate the significant difference of two groups of data in all experiments. The data were expressed as the mean ± standard deviation (SD) for at least three independent experiments. Differences were considered significantly when *P* < 0.05.

## RESULTS

### URGCP/URG4 is upregulated in bladder cancer cells and tissues

**Figure 1:**
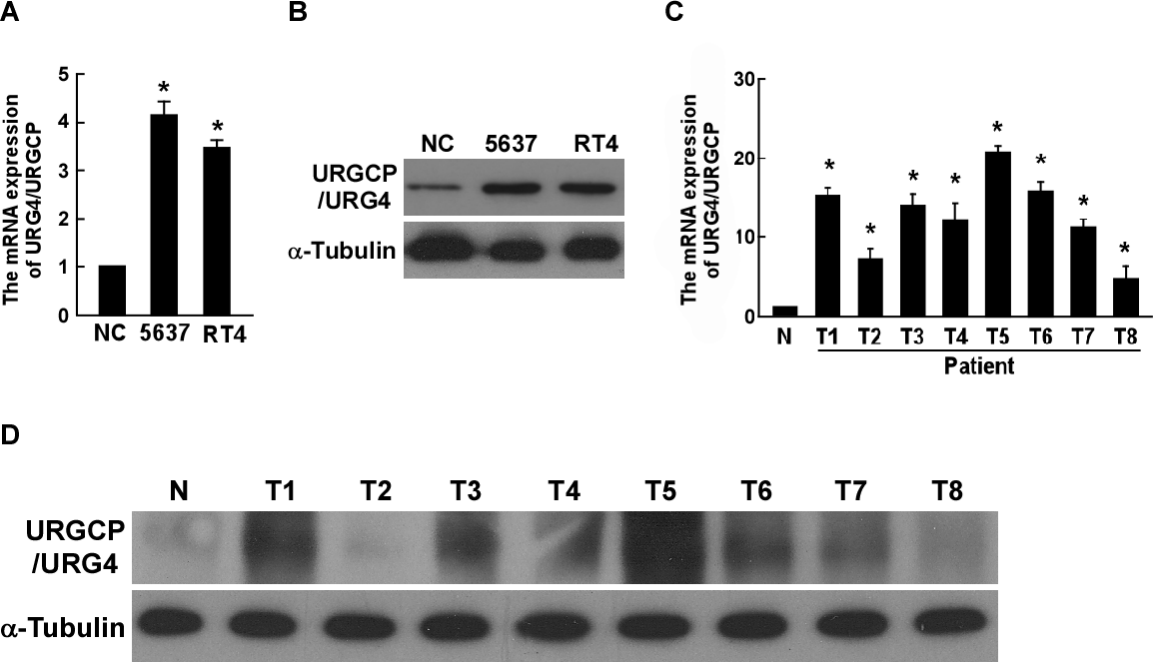
URGCP/URG4 is upregulated in bladder cancer cells and tissues **A.** Quantitative real-time PCR determined *URGCP/URG4* mRNA expression in bladder cancer cell lines, 5637 and RT4; NC indicated primary-cultured normal bladder epithelial cells, *GAPDH* was used as control. **B.** Western blotting assay determined URGCP/URG4 expression in bladder cancer cell lines, 5637 and RT4, determined by western blotting assay, α-tubulin was used as control. **C.** Quantitative real-time PCR determined *URGCP/URG4* mRNA expression in eight fresh bladder cancer tissues and one normal bladder tissue; *GAPDH* was used as control. **D.** Western blotting assay determined URGCP/URG4 expression in eight fresh bladder cancer tissues, and one normal bladder tissue, α-tubulin was used as control. Each bar represents the mean ± SD of three independent experiments. *P < 0.05.

To determine URGCP/URG4 expression in bladder cancer cells and tissues, qRT-PCR and western blotting assay showed that both mRNA and protein expression of URGCP/URG4 were significantly upregulated in bladder cancer cells, compared to that of in the primary-cultured normal bladder epithelial cells (indicated as NC) (Figure 1A and 1B). Meanwhile, we used eight fresh bladder cancer tissues and one normal bladder tissue to investigate URGCP/URG4 expression, and found URGCP/URG4 expression was upregulated in most of bladder cancer tissues (indicated as T) compared to that of in normal tissue (indicated as N) (Figure 1C and 1D). These suggested that URGCP/URG4 is upregulated in bladder cancer, indicating a putative correlation with bladder cancer progression.

### Ectopic URGCP/URG4 inhibits cisplatin-induced apoptosis of bladder cancer cells

**Figure 2:**
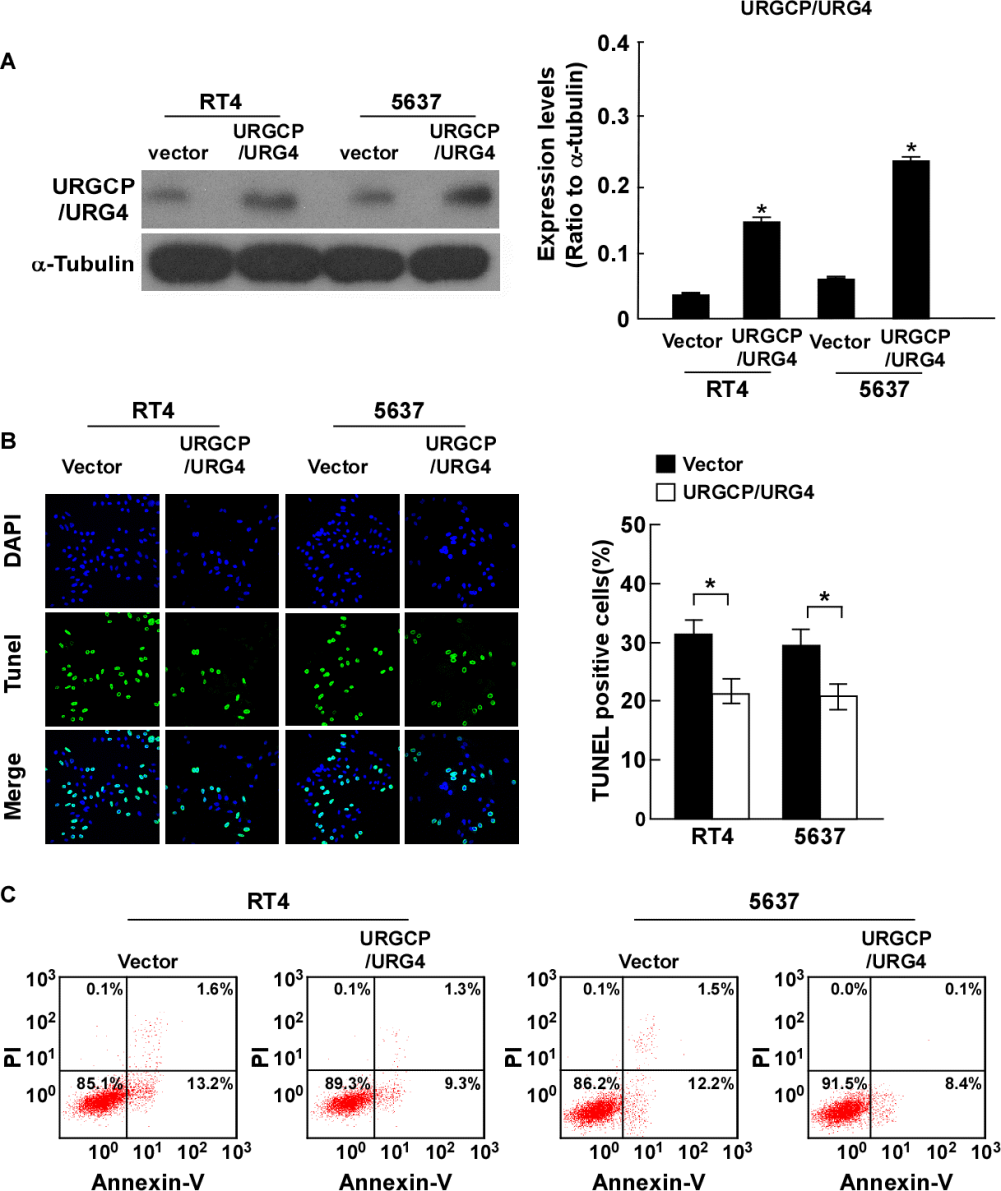
Overexpression of URGCP/URG4 inhibits cisplatin-induced apoptosisof bladder cancer cells **A.** Western blotting analysis confirmed URGCP/URG4 expression in 5637 and RT4 cells stably overexpressing URGCP/URG4, α-tubulin was used as control. **B.** Ectopic expression of URGCP/URG4 inhibited cisplatin-induced apoptosis determined by TUNEL assay. Left panel, immunofluorescent images of TUNEL stained cells of indicated cells treated with 40g/ml cisplatin for 24 hours. Right panel, quantification of TUNEL stained cells. TUNEL staining-positive cells were counted from 10 random fields after indicated cells. **C.** Annexin V/PI double staining was performed to measure the apoptosis ratio in indicated cells. Representative flow cytometry profiles and percentages of apoptotic cells (Annexin V-positive and PI-negative cells in lower right quadrant) are shown. Each bar represents the mean ± SD of three independent experiments. *P < 0.05.

To explore whether upregulation of URGCP/URG4 promotes the resistance chemotherapeutics-induced apoptosis of bladder cancer, RT4 and 5637 cells stably overexpressing URGCP/URG4 were established for further study. The ectopic expression of URGCP/URG4 in both stable cell lines was confirmed by western blotting assay (Figure 2A). We examined the effect of URGCP/URG4 on chemotherapeutics-induced cell apoptosis, by exposing these cells to anti-tumor agent cisplatin. Cisplatin is a well-known and popularity chemotherapeutic drug, it kill cancer cells by causing DNA damage and initiating apoptosis [1].

TUNEL assay found the apoptosis cells were significantly fewer in indicated URGCP/URG4-overexpressing cells (Figure 2B). Annexin-V binding assays also showed the number of Annexin-V-FITC-positive cells in indicated URGCP/URG4-overexpressing cells was decreased when cisplatin was used (Figure 2C). All the results revealed that ectopic URGCP/URG4 inhibited cisplatin-induced apoptosis, it might play critical role in bladder cancer progression and therapy.

### Inhibition of URGCP/URG4 enhances cisplatin-induced apoptosis of bladder cancer cells

**Figure 3:**
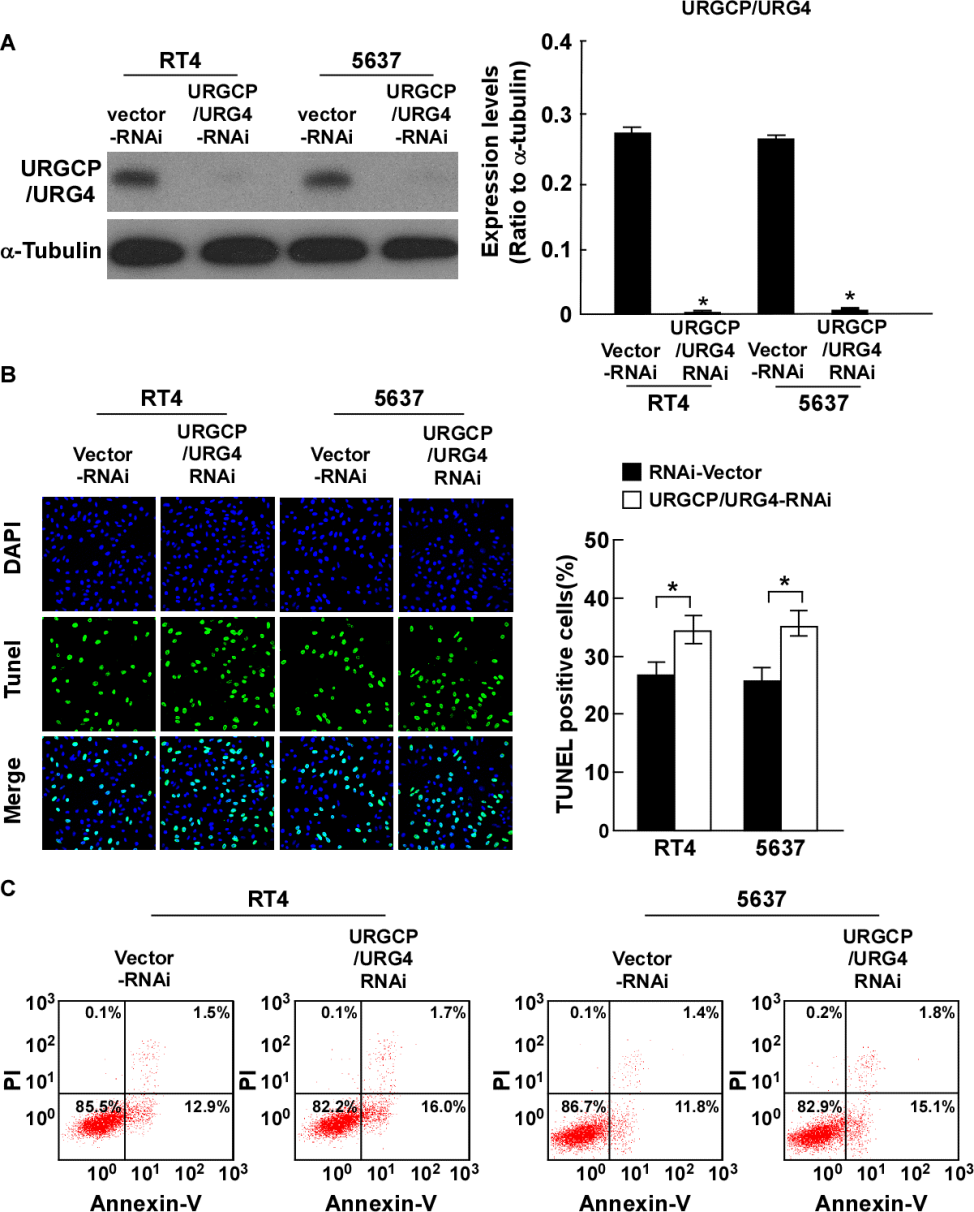
Silencing URGCP/URG4 promotes cisplatin-induced apoptosis ofbladder cancer cells **A.** Western blotting analysis determined URGCP/URG4 expression in 5637 and RT4 cells stably silencing URGCP/URG4, α-tubulin was used as control. **B.** Silencing URGCP/URG4 promoted cisplatin-induced apoptosis determined by TUNEL assay. Left panel, immunofluorescent images of TUNEL stained cells of indicated cells treated with 40g/ml cisplatin for 24 hours. Right panel, quantification of TUNEL stained cells. TUNEL staining-positive cells were counted from 10 random fields after indicated cells. **C.** Annexin V/PI double staining was performed to measure the apoptosis ratio in indicated cells. Representative flow cytometry profiles and percentages of apoptotic cells (Annexin V-positive and PI-negative cells in lower right quadrant) were shown. Each bar represents the mean ± SD of three independent experiments. *P < 0.05.

To further confirm the function of URGCP/URG4 on cisplatin-induced apoptosis, we downregulated URGCP/URG4 expression using RNA inference. The effects of URGCP/URG4 knockdown in indicated cells were confirmed by western blotting assay (Figure 3A). URGCP/URG4 was decreased in indicated cells transfected with URGCP/URG4 shRNA and could be rescued by URGCP/URG4 overexpression (Supplemental Figure 1). Both the TUNEL and Annexin-V binding assays found URGCP/URG4 silencing cell lines had less resistance to cisplatin-induced apoptosis, and the number of apoptotic cells was significantly higher than that in empty vector control cells (Figure 3B and 3C). Taken together, our results suggested that knockdown of URGCP/URG4 impaired the ability of bladder cancer cells to resist cell death induced by cisplatin.

### URGCP/URG4 inhibits cisplatin-induced apoptosis of bladder cancer cells *in vivo*

**Figure 4:**
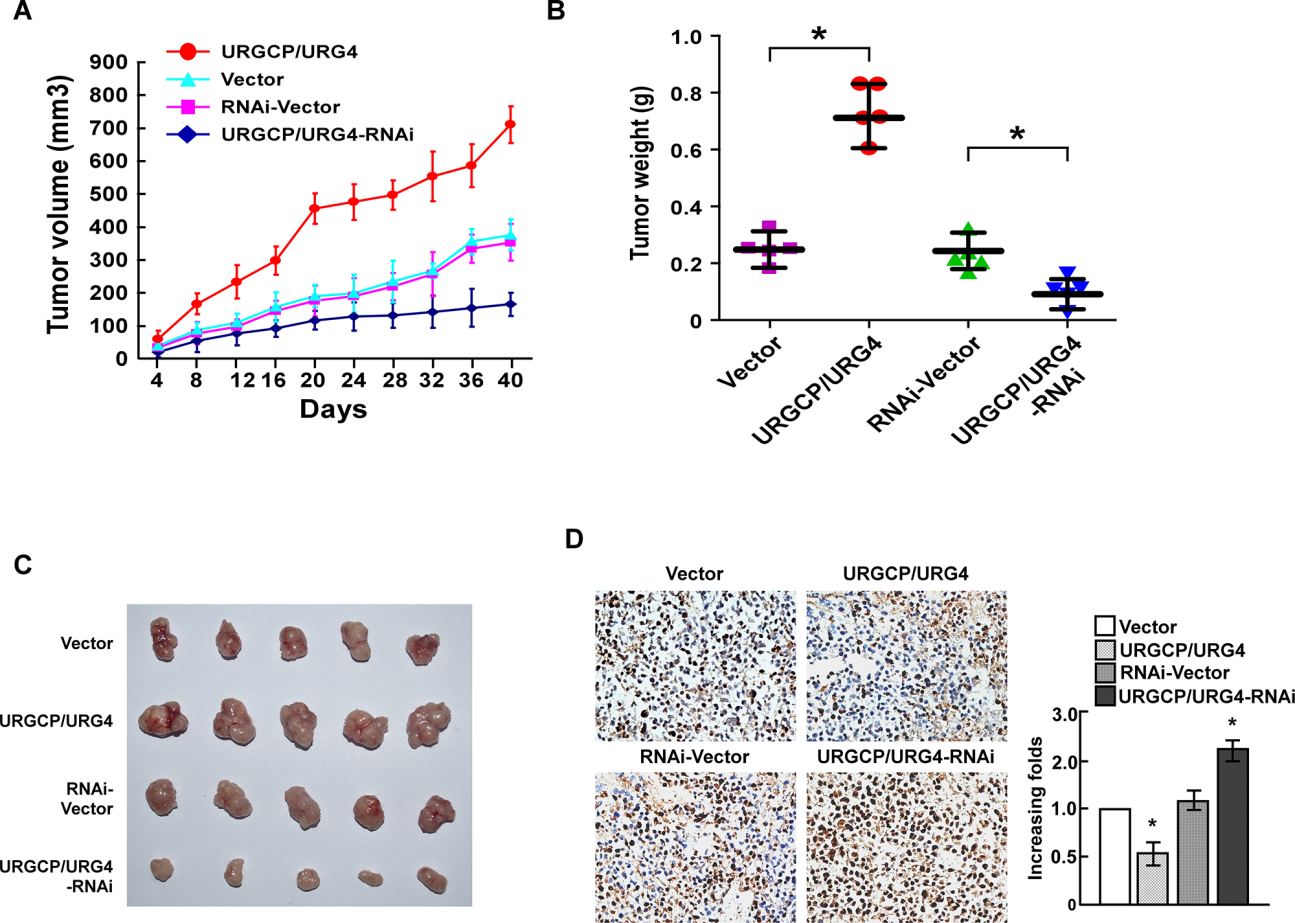
URGCP/URG4 inhibits cisplatin-induced apoptosis of bladder cancercells *in vivo* **A.** Average tumor volumes (mm^3^) of different treatment groups after inoculation. **B.** Analysis of weight of subcutaneous tumors from the different treatment groups. **C.** Representative images of tumors in the different treatment groups. **D.** Apoptosis cells determined by TUNEL assay in different treatment groups. Bars represent the means ± SD of three independent experiments. *P < 0.05.

To further assess the effect of URGCP/URG4 on resistance to cisplatin-induced apoptosis, we determined its function *in vivo* by inoculating nude mice with tumor cells. After treated with cisplatin, the volume and weight of URGCP/URG4-overexpressing tumors were markedly higher compared to the tumors formed by control cells, indicating the anti-apoptosis function of URGCP/URG4 on bladder cancer cells *in vivo* (Figure 4A–4C). We also used the stable URGCP/URG4-silencing cells to confirm the function *in vivo*. Consistently, inhibition of URGCP/URG4 enhanced cisplatin-induced apoptosis of bladder cancer cells, for which tumor sizes were much smaller (Figure 4A–4C). To investigate apoptosis of tumor xenografts, TUNEL assay was used. Tumors from URGCP/URG4-overexpressing xenografts displayed fewer apoptotic cells, while tumors from URGCP/URG4-silencing xenografts showed more apoptotic cells, than that in the control groups (Figure 4D). Taken together, these results demonstrated that URGCP/URG4 inhibits cisplatin-induced apoptosis of bladder cancer cells *in vivo*.

### URGCP/URG4 regulates the expression of apoptosis related proteins and activates NF-κB

**Figure 5:**
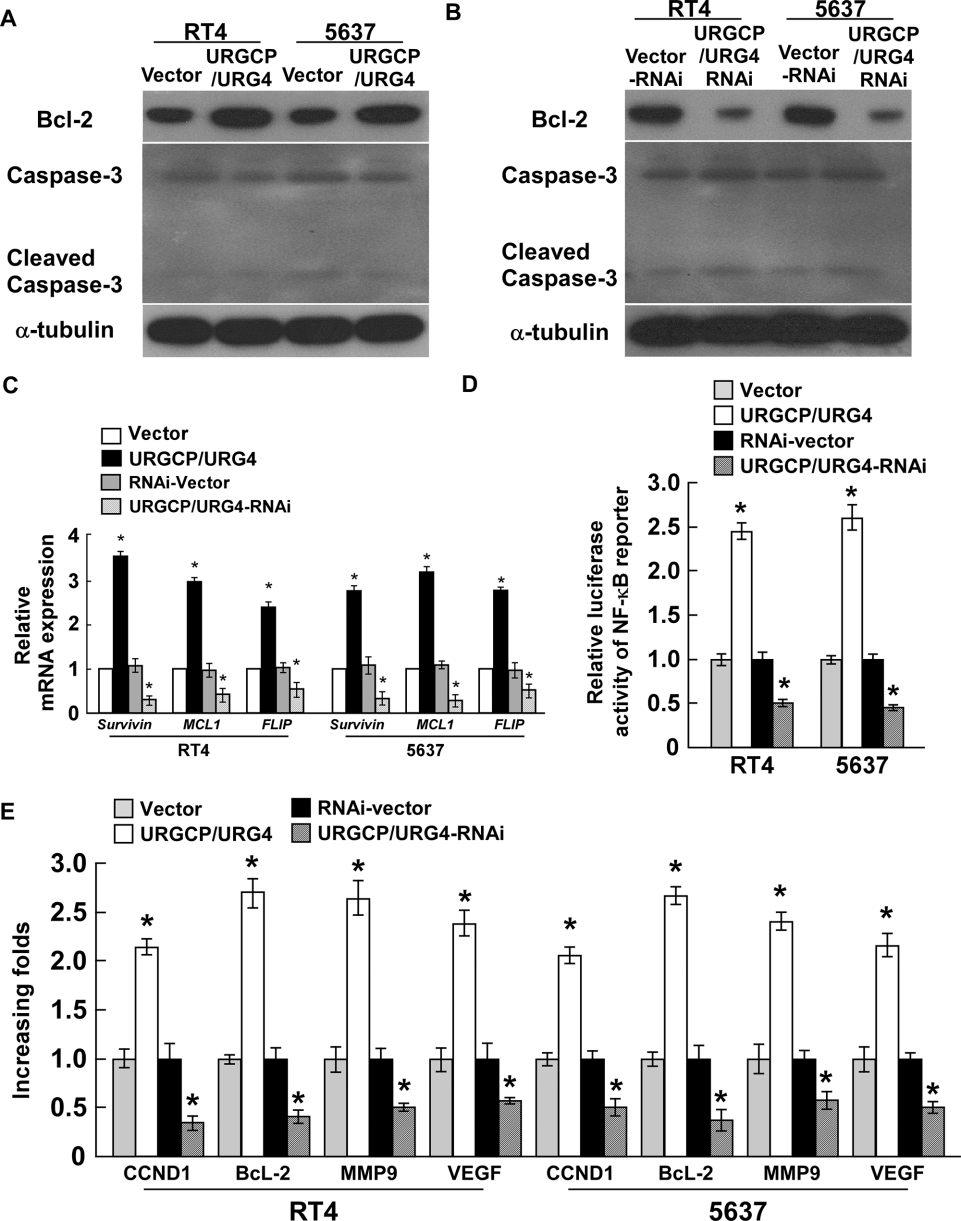
URGCP/URG4 regulates the expression of apoptotic related proteinsand activates NF-κB **A.** Western blotting determined Bcl-2, Caspase-3 and Cleaced Caspase-3 expression in URGCP/URG4 overexpressing cells, α-tubulin was used as control. **B.** Western blotting analyzed Bcl-2 and Caspase 3 expression in URGCP/URG4 silencing cells, α-tubulin was used as control. **C.** The expression of anti-apoptotic factors, Survival, MCL1 and FLIP when URGCP/URG4 was overexpressed or knock-downed. **D.** The transcriptional activity of NF-κB determined by the luciferase assay when URGCP/URG4 was upregulated or downregulated. **E.** The expression of NF-κB targeted genes,*CCND1*, *BCL-2*, *MMP9* and *VEGF*, determined by real-time PCR, *GAPDH* was used as control. Each bar represents the mean ± SD of three independent experiments. *P < 0.05

To investigate whether URGCP/URG4 regulates apoptosis related proteins, we examined the expression of anti-apoptotic protein Bcl-2, and Caspase-3 which are activated in the apoptotic cells when URGCP/URG4 was overexpressed or knock-downed. The results showed that URGCP/URG4 overexpression increased Bcl-2 expression and decreased Caspase-3 and Cleaved-Caspase-3 expression (Figure 5A). When URGCP/URG4 was knock-downed, Bcl-2 was downregulated, Caspase-3 and Cleaved Caspase-3 were upregulated (Figure 5B). We also determined Survival, mcl-1 and FLIP expression, which all act as antipoptotic proteins. We found URGCP/URG4 overexpression significantly promoted these genes expression, knockdown of URGCP/URG4 inhibited these genes expression (Figure 5C). these suggested URGCP/URG4 is an anti-apoptotic facilitator.

NF-κB as an anti-apoptotic transcription factor, have been widely studied, activation of NF-κB induces many anti-apoptotic factors, such as cIAPs, FLICE, FLIP, A1/BFL1 and Bcl-X_L_ [12, 13]. We determined the activity of NF-κB using a luciferase reporter system. The results showed ectopic URGCP/URG4 promoted NF-κB transcriptional activity, silencing URGCP/URG4 inhibited the activity of NF-κB transcription (Figure 5D). qRT-PCR analysis found the expression of NF-κB-targeted genes, including *CCND1*, *Bcl-2*, *MMP9* and *VEGF* were upregulated once overexpression of URGCP/URG4, whereas knockdown of URGCP/URG4 significantly inhibited the levels of these genes in bladder cancer cells (Figure 5E). Survival, MCL-1 and FLIP also are the targets of NF-κB pathway, we found URGCP/URG4 enhances their expression. these suggested URGCP/URG4 might regulate the resistance to cisplatin-induced apoptosis by activating NF-κB pathway.

**Figure 6:**
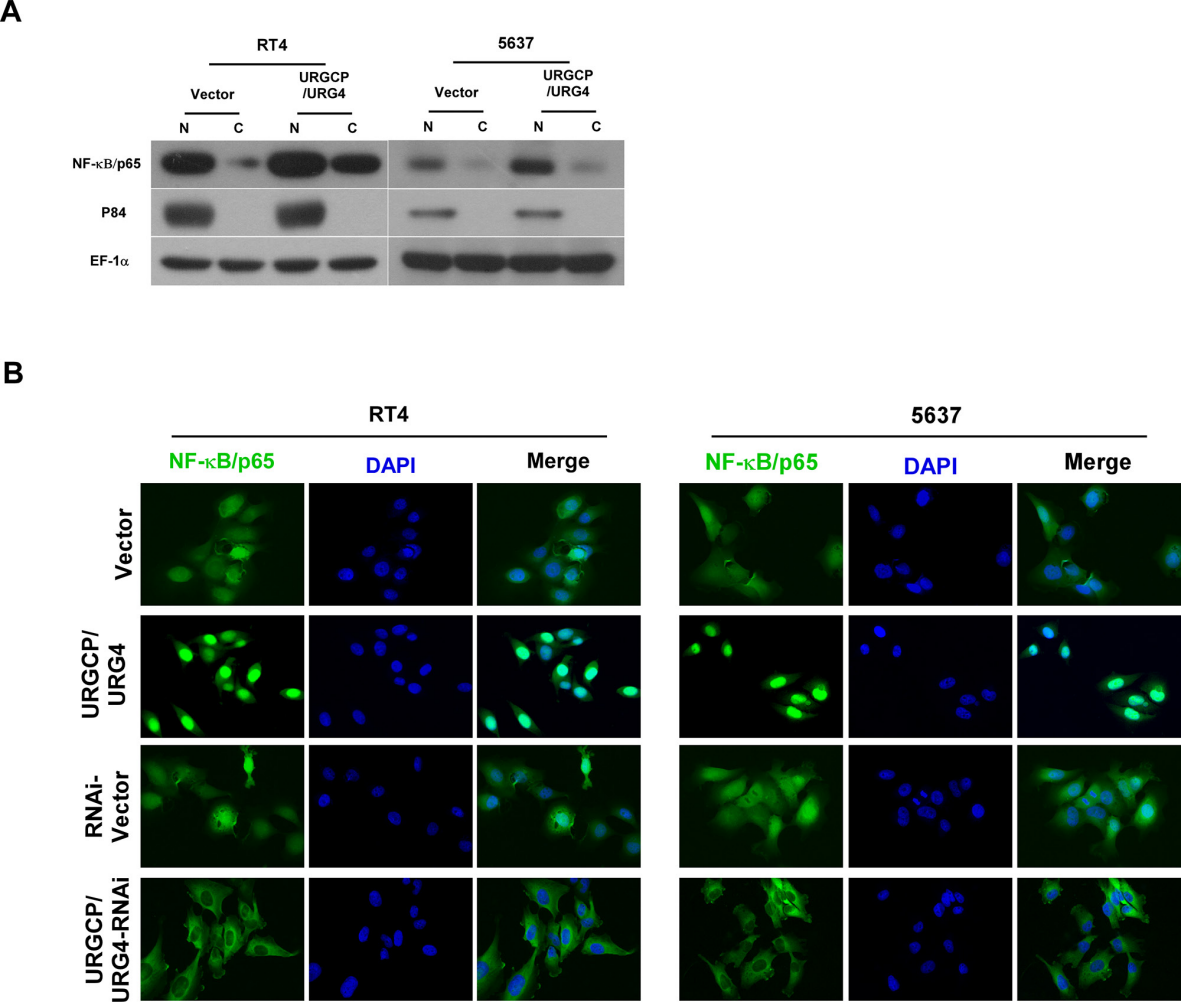
URGCP/URG4 activates NF-κB pathway **A.** Western blotting analyzed the location of p65 when URGCP/URG4 was upregulated, p84 was used as a positive control, EF-1α was used a loading control. **B.** Immunofluorescence assay determined the location of p65 when URGCP/URG4 was upregulated or downregulated. DAPI was used to indicate nucleus.

To investigate this speculation, we further examined whether URGCP/URG4 could activate NF-κB. The marker of NF-κB activation is that RelB (p65)-p52 dimers translocate to the nucleus, so we determined the location of p65 by modulating URGCP/URG4 expression. We separated nuclear and cytoplasmic proteins used KeyGEN Nuclear and Cytoplasmic Protein Extraction Kit (KeyGEN, China) according to the manufacture's instruction. Western blotting showed that p65 expression was significantly increased in the nucleus of indicated cells after URGCP/URG4 overexpression, nuclear matrix protein p84 was used to indicate protein expression in the nucleus (Figure 6A). We further confirmed this result used immunofluorescence, when URGCP/URG4 was upregulated in indicated cells, p65 translocated to the nucleus, it mainly expressed in nucleus. When URGCP/URG4 was downregulated, it mainly expressed in cytoplasmic (Figure 6B). these results suggested URGCP/URG4 could activate NF-κB pathay.

All the results revealed that URGCP/URG4 promotes the resistance to cisplatin-induce apoptosis in bladder cancer cells via activation of NF-κB signaling pathway.

### Correlation between URGCP/URG4 expression and bladder cancer clinicopathplogic characteristics

**Figure 7:**
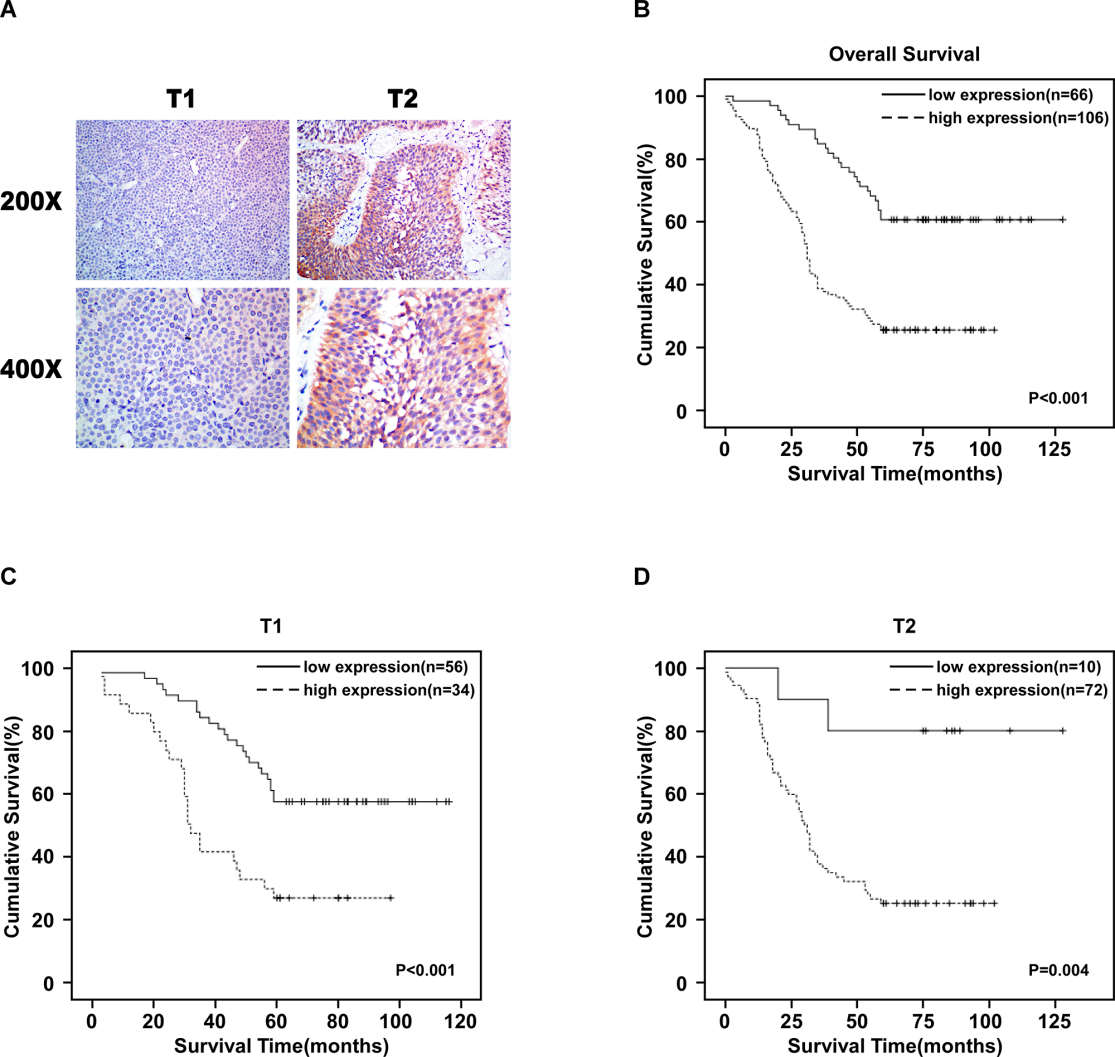
URGCP/URG4 is upregulation in bladder cancer patients’ tissues and correlated with poor clinical outcome **A.** Representative images of URGCP/URG4 expression in T1 and T2 tumors determined by IHC (magnification: 200 ×, 400 ×). **B.** Kaplan-Meier analysis for overall survival. *p* < 0.001. **C.** Kaplan-Meier analysis for overall survival in patients in T1 stage. *p* < 0.001. **D.** Kaplan-Meier analysis for overall survival in patients in T2 stage. *P* = 0.04.

To investigate the clinic significance of URGCP/URG4 expression. we investigated the correlation between URGCP/URG4 expression and the patients’ clinicopathplogic characteristics used 127 paraffin-embedded, archival bladder cancer tissues. The tissues included 90 T1 tumors and 82 T2 tumors. IHC analysis revealed URGCP/URG4 was high expression in 106 (61.6%) cases, and low expression in 66 (38.4%) cases (Table 1), and its expression advanced with larger tumor size (T classification) (Figure 7A), the detailed date were shown as follow: 37.8% for T1 (34/90), 87.8% for T2 (72/82). These suggested URGCP/URG4 is upregulated advanced with T classification.

Our analysis revealed the expression of URGCP/URG4 was significantly correlated with degree of differentiation (*p* < 0.001), larger tumor size (*p* < 0.001), lymph node involvement (N classification) (*p* < 0.001), short survival time (*p* < 0.001) and vital status (*p* < 0.001). These observation suggested a positive correlation between URGCP/URG4 expression and clinical progression in bladder cancer. However, no evident correlations were observed between URGCP/URG4 expression and age, gender, tumor number recurrence (Table 2).

**Table 2:** Correlation between URG4 expression and clinicopathologic characteristics of bladder cancer patient

Characteristics		Total	URG4		chi-square test *P*-value
			Low expression	High expression	
Age(y)	<64	81	30	51	0.756
	≥64	91	36	55	
Degree of differentiation	I	32	23	9	<0.001
	II	53	25	28	
	III	87	18	69	
T classification	T_1_	90	56	34	<0.001
	T_2_	82	10	72	
N classification	0	150	66	0	<0.001
	1	22	84	22	
Gender	Male	157	60	97	0.549
	Female	15	6	9	
Number	<3	108	41	67	0.794
	≥3	64	25	39	
Size	<3	72	35	37	0.026
	≥3	100	31	69	
Recurrence	Yes	90	29	61	0.087
	No	82	37	45	
Vital status (at follow-up)	alive	67	40	27	<0.001
	Dead	105	26	79	

we further confirmed the correlation between URGCP/URG4 expression and the patients’ clinicopathplogic characteristics used Spearman correction analysis. Our analysis revealed the expression of URGCP/URG4 was significantly correlated with degree of differentiation (*p* < 0.001), larger tumor size (*p* < 0.001), lymph node involvement (*p* < 0.001), short survival time (*p* < 0.001) and vital status(*p* < 0.001). These observation suggested a correlation between increased URGCP/URG4 expression and clinical progression in bladder cancer. However, no evident correlations were shown between URGCP/URG4 expression and other features, including age, gender and recurrence (Table 3).

**Table 3:** Spearman analysis of correlation between URG4 and clinicopathological characteristics

Variables	C14ORF166 expression level	
	Spearman Correlation	*p*-Value
Survival time	−0.477	<0.001
Vital status	0.350	<0.001
Age	−0.026	0.736
Size	0.179	0.019
Number	−0.011	0.887
Degree of differentiation	0.604	<0.001
T classification	0.514	<0.001
N classification	0.302	<0.001
Gender	−0.010	0.893
Recurrence	0.132	0.083

### High URGCP/URG4 expression in primary bladder cancer tissues correlates with poor patient survival

To investigate the relationship between URGCP/URG4 expression and clinical outcome, we determined the correlation between URGCP/URG4 expression and patient survival. Kaplan-Meier survival curves demonstrated the overall survival of patients with high expression of URGCP/URG4 was significantly shorter than those with low URGCP/URG4 expression (Figure 7B, *p* < 0.001). We also found high expression of URGCP/URG4 was significantly shorter than those with high URGCP/URG4 expression in patients which were classified into T1 (*p* < 0.001) or T2 (*p* = 0.004) (Figure 7C and 7D). these suggested high URGCP/URG4 expression in primary bladder cancer patients with poor survival.

In addition, we used Cox-regression analysis to determine whether URGCP/URG4 could serve as a useful prognostic factor. As shown in Table 4, we found URGCP/URG4 expression was a independent prognostic factor for bladder cancer patients. These suggested the expression of URGCP/URG4 is correlated with the prognosis of bladder cancer significantly.

**Table 4:** Univariate and multivariate analyses of various prognostic parameters in patients with bladder cancer Cox-regression analysis

	Univariate analysis			Multivariate analysis		
	No. patients	*p*	Regression coefficient (SE)	*p*	Relative risk	95% confidence interval
**URG4**		<0.001	1.202(0.246)	<0.001	3.403	1.976–5.859
Low expression	66					
High expression	106					
**T classification**		0.038	0.442(0.213)	0.313	0.775	0.472–1.271
T_1_	90					
T_2_	82					
**N classification**		<0.001	1.199(0.326)	0.014	2.361	1.189–4.688
0	150					
1	22					

## DISCUSSION

In our study, we demonstrated that URGCP/URG4 was upregulated in bladder cancer cells and tissues. Ectopic expression of URGCP/URG4 promoted the resistance to cisplatin-induced cell apoptosis in bladder cancer, while silencing expression of URGCP/URG4 enhanced the cisplatin-induced apoptosis, both *in vitro* and *in vivo*. In addition, ectopic URGCP/URG4 promoted anti-apoptotic factor expression and inhibited pro-apoptotic factor expression. We further analysis found URGCP/URG4 could activate NF-κB pathway whose target genes are critical for apoptosis, these suggested URGCP/URG4 promoted cisplatin-induced apoptosis by activating NF-κB pathway. We also analyzed the correlation between URGCP/URG4 expression and clinical parameters, and found URGCP/URG4 expression was correlated with bladder cancer progression, it was an unfavorable factor for patient's survival.

The chemotherapy is one of the most widely used and effective methods for tumors treatment [14]. However, Drug resistance remains a major clinical challenge and great obstacle for current cancer therapy [15]. The mechanism of drug resistance in cancer chemotherapy is complicated and unclear. The death of tumor cells induced by chemotherapy is largely mediated by activation of apoptosis, while inhibition of apoptosis will increase tumor cells resistant to anti-tumor treatment [16]. Multiple genes related to apoptosis resistance have been found to contribute to drug resistance. For example, overexpression of survivin promotes cancer cell growth and drug resistance in multiple types of cancers [17]. Specific inhibition of the survival signaling resulted in a significant enhancement of paclitaxel-induced apoptosis [18]. It was reported that Bmi-1 upregulation protects glioma cells from apoptosis induced by anticancer drugs doxorubicin [19]. In our study, we determined that URGCP/URG4 overexpression promoted the resistance to cisplatin-induced bladder cancer cells apoptosis, indicating its function and potential utilization in bladder cancer chemotherapeutic treatment. It has found that some molecular dysregulation has the potential to cause apoptotic dysregulation, including activation of anti-apoptotic factor, such as Bcl-2, Bcl-xL [20], inactivation of pro-apoptotic effectors, such as p53 and Caspases [21], or induction of survival signals including survivin, FLIP, and NF-κB [22]. The genes modulating drug resistance also regulate the apoptotic related factors. For examples, Survivin enhances drug resistance via binding the effectors cell death proteases Caspases and inhibiting Caspases activity [23]. Bmi-1 is reported to promote apoptotic resistance via activating NF-κB and inducing expression of NF-κB-targeted genes [19]. Our study confirmed that URGCP/URG4 overexpression enhanced anti-apoptotic factor expression, such as Bcl-2 and FLIP, and inhibited pro-apoptotic factor expression, such as Caspase-3. We further analyzed the mechanism and found URGCP/URG4 activated NF-κB pathway, it could promote p65 translocate to the nucleus, which a marker for activating NF-κB pathway. NF-κB pathway plays an important role in regulating cancer cells proliferation, anti-apoptosis, invasion and metastasis and angiogenesis, through modulating series of functional genes expression and activity, including IL1A, CCND1, MYC, Bcl-2, MMP9 and VEGF [22, 24]. We also found URGCP/UGR4 overexpression promoted Bcl-2, CCND1, MMP9 and VEGF expression, this further confirmed URGCP/URG4 could activated NF-κB pathway.

We also analyzed the correlation between URGCP/URG4 expression and clinicopathologic parameters, and found high URGCP/URG4 expression was correlated with advanced clinicopathologic classifications (T and N), high expression of URGCP/URG4 indicated an unfavorable overall survival and served as a high risk marker of bladder cancer, these suggested URGCP/URG4 is an unfavorable prognostic factors. But the further mechanism of URGCP/URG4 regulating drug resistance needed to be investigate further. Cancer stem cell plays critical role in drug resistance [25], whether URGCP/URG4 could regulate bladder cancer stem cell still needed to be investigated.

In summary, we found URGCP/URG4 was high expression in bladder cancer cells and tissues, it promoted the resistance to cisplatin-induced cell apoptosis in bladder cancer, mechanism analysis found it activated NF-κB pathway to inhibit apoptosis. The correlation analysis between URGCP/URG4 expression and clinicopathologic parameters suggested URGCP/URG4 expression was correlated with the progression of bladder cancer, and was an unfavorable prognostic factor.

## SUPPLEMENTARY FIGURE


