# Supplementary material for: URGCP/URG4 promotes apoptotic resistance in bladder cancer cells by activating NF-κB signaling
Source: Oncotarget. 2015 Sep 2;6(31):30887–901. doi: 10.18632/oncotarget.5134 (PMC4741575; doi:10.18632/oncotarget.5134)
Supplement: Supplementary file 1 [file oncotarget-06-30887-s001.pdf]

SUPPLEMENTARY FIGURE

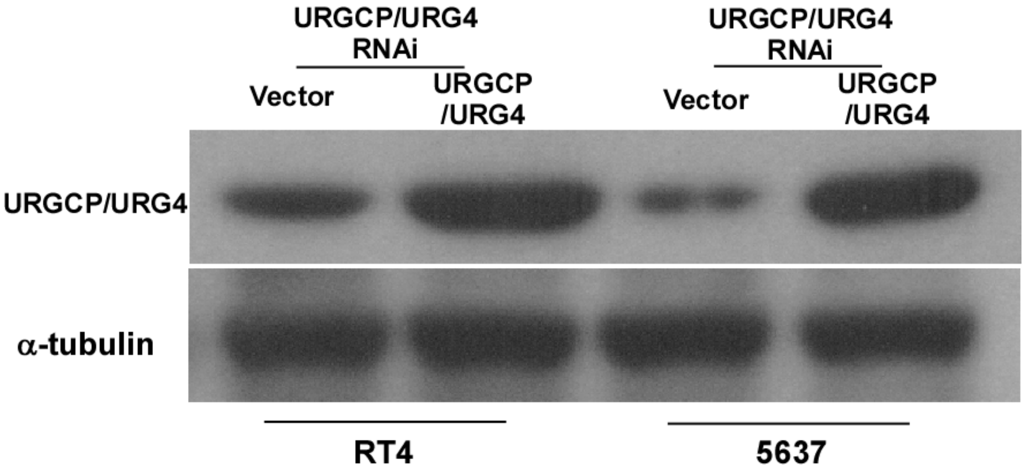

Supplementary Figure S1: Western blotting determined the off-target effect of URGCP/URG4 shRNA. URGCP/URG4 was knock-downed in indicated cells transfected with URGCP/URG4 shRNA and could be rescued by URGCP/URG4 overexpression, α-tubulin was used as control.
